# Supplementary material for: YAP and TAZ regulate adherens junction dynamics and endothelial cell distribution during vascular development
Source: eLife. 2018 Feb 5;7:e31037. doi: 10.7554/eLife.31037 (PMC5814147; doi:10.7554/eLife.31037)
Supplement: Supplementary file 1. [file elife-31037-supp1.docx]

| Reagent | Reference | Company | Final conc. | Duration of treatment: Luciferase reporter assay/  Scratch wound assay/  Permeability assay/  VE-Cadherin staining | Literature reference |
| --- | --- | --- | --- | --- | --- |
| DBZ | 14627 | Cayman chemicals | 0.1uM | 20hrs/16hrs | (1) |
| Recombinant-hGremlin | 5190-GR | R&D Systems |  0.1ug/ml | 20hrs | (2, 3) |
| Recombinant-hEndoglin | 1097-EN | R&D Systems | 0.25ug/ml | 20hrs | (4) |
| LDN-193189 | 19396 | Cayman chemicals | 1uM | 20hrs/16hrs/24h/24h | (5) |
| K02288 | 16678 | Cayman chemicals | 1uM | 20hrs | (5) |
| Recombinant hAlk1fc | 370-AL-100 | R&D Systems | 25ng/ml | 20hrs | (6, 7) |

**Supplementary table 1**. List of reagents used to manipulate Notch and BMP signaling in cell culture.

1. Ridgway J, Zhang G, Wu Y, Stawicki S, Liang WC, Chanthery Y, Kowalski J, Watts RJ, Callahan C, Kasman I, et al. Inhibition of Dll4 signalling inhibits tumour growth by deregulating angiogenesis. *Nature.* 2006;444(7122):1083-7.

2. Mitola S, Ravelli C, Moroni E, Salvi V, Leali D, Ballmer-Hofer K, Zammataro L, and Presta M. Gremlin is a novel agonist of the major proangiogenic receptor VEGFR2. *Blood.* 2010;116(18):3677-80.

3. Grillo E, Ravelli C, Corsini M, Ballmer-Hofer K, Zammataro L, Oreste P, Zoppetti G, Tobia C, Ronca R, Presta M, et al. Monomeric gremlin is a novel vascular endothelial growth factor receptor-2 antagonist. *Oncotarget.* 2016;7(23):35353-68.

4. Castonguay R, Werner ED, Matthews RG, Presman E, Mulivor AW, Solban N, Sako D, Pearsall RS, Underwood KW, Seehra J, et al. Soluble endoglin specifically binds bone morphogenetic proteins 9 and 10 via its orphan domain, inhibits blood vessel formation, and suppresses tumor growth. *J Biol Chem.* 2011;286(34):30034-46.

5. Sanvitale CE, Kerr G, Chaikuad A, Ramel MC, Mohedas AH, Reichert S, Wang Y, Triffitt JT, Cuny GD, Yu PB, et al. A new class of small molecule inhibitor of BMP signaling. *PLoS One.* 2013;8(4):e62721.

6. Mitchell D, Pobre EG, Mulivor AW, Grinberg AV, Castonguay R, Monnell TE, Solban N, Ucran JA, Pearsall RS, Underwood KW, et al. ALK1-Fc inhibits multiple mediators of angiogenesis and suppresses tumor growth. *Mol Cancer Ther.* 2010;9(2):379-88.

7. Larrivee B, Prahst C, Gordon E, del Toro R, Mathivet T, Duarte A, Simons M, and Eichmann A. ALK1 signaling inhibits angiogenesis by cooperating with the Notch pathway. *Dev Cell.* 2012;22(3):489-500.
