# Supplementary material for: YAP and TAZ regulate adherens junction dynamics and endothelial cell distribution during vascular development
Source: eLife. 2018 Feb 5;7:e31037. doi: 10.7554/eLife.31037 (PMC5814147; doi:10.7554/eLife.31037)
Supplement: Supplementary file 2. [file elife-31037-supp2.docx]

|  | Reference | Dilution | Company |  |
| --- | --- | --- | --- | --- |
| Yap | 46189 | 1:100 | ThermoFisher Scientific | Retinas + HUVECs |
| Taz | HPA007415 | 1:100 | Sigma | Retinas + HUVECs |
| Erg | sc-18136 | 1:100 | Santa Cruz Biotechnology | Retinas |
| Erg | Ab92513 | 1:1000 | Abcam | Retinas |
| VE-Cadherin | 555289 | 1:100 | BD Biosciences | Retinas |
| TER-119 | MAB1125 | 1:100 | R&D Systems | Retinas |
| PECAM-1 | AF3628 | 1:200 | R&D Systems | Retinas |
| Cleaved Caspase 3 | AF835 | 1:200 | R&D Systems | Retinas |
| Dll4 | AF1389 | 1:100 | R&D Systems | Retinas |
| pSMAD1/5/8 | 13820S | 1:1000 | Cell Signalling | Retinas |
| Phalloidin- Alexa-Fluor 488 | A12379 | 1:100 | ThermoFisher Scientific | HUVECs |
| Ib4-Alexa-Fluor 647 Conjugate | I32450 | 1:1000 | ThermoFisher Scientific | Retinas + HUVECs |
| Ib4-Alexa-Fluor 488 Conjugate | I21411 | 1:1000 | ThermoFisher Scientific | Retinas + HUVECs |
| Ib4-Alexa-Fluor 568 Conjugate | I21412 | 1:1000 | ThermoFisher Scientific | Retinas + HUVECs |

**Supplementary table 2**. List of primary antibodies and dyes used.
