# Supplementary material for: YAP and TAZ regulate adherens junction dynamics and endothelial cell distribution during vascular development
Source: eLife. 2018 Feb 5;7:e31037. doi: 10.7554/eLife.31037 (PMC5814147; doi:10.7554/eLife.31037)
Supplement: Supplementary file 3. [file elife-31037-supp3.docx]

| Target | Assay ID |
| --- | --- |
| LFNG | Hs00385436_g1 |
| DLL4 | Hs00184092_m1 |
| NRARP | Hs04183811_s1 |
| Hes1 | Hs00172878_m1 |
| Hey1 | Hs01114113_m1 |
| SMAD6 | Hs00178579_m1 |
| ENG | Hs00923996_m1 |
| UNC5B | Hs00900710_m1 |
| ID1 | Hs03676575_s1 |
| ID3 | Hs00171409_m1 |
| CTGF | Hs00170014_m1 |
| CYR61 | Hs00998500_g1 |
| ANKRD1 | Hs00923599_m1 |
| INHBA1 | Hs01081598_m1 |
| GAPDH | Hs02786624_g1 |
| HPRT1 | Hs02800695_m1 |
| ACTB | Hs99999903_m1 |

**Supplementary table 3**. List of the TaqMan primers (Applied Biosystems) used.
